# A case report on a large, peduncular intra‐abdominal hepatocellular carcinoma extending into the retroperitoneum

**DOI:** 10.1002/ccr3.3751

**Published:** 2021-01-08

**Authors:** Dana M. Omer, Jordan Dozier, Zongxian Cao, Hongfa Zhu, Donald A. McCain

**Affiliations:** ^1^ Hackensack University Medical Center Hackensack NJ USA

**Keywords:** gastrectomy, HCC, hepatectomy, hepatitis, Hepatocellular carcinoma, histology, pathology, surgical oncology

## Abstract

Here, we discuss a relatively uncommon presentation of a hepatocellular carcinoma and discuss its preoperative planning and surgical intervention required to reach complete resection.

## Graphical abstract

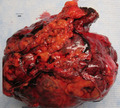

## 1 BACKGROUND

Hepatocellular carcinoma is a primary liver malignancy often caused by viral hepatitis infection due to chronic inflammation and persistent cytokine release. Although rare, patients may present with large, pedunculated hepatocellular neoplasms that extend into the intra‐abdominal space. Here, we present a case report on a 66‐year‐old male who was found to have such a mass that required careful pre‐operative planning to complete a challenging, yet worthwhile, surgical intervention with R0 resection. A 66‐year‐old Caucasian male with a history of Hepatitis C presented with 4‐5 months of abdominal pain, distention, and weight loss. Imaging revealed a large heterogenous mass attached to the left lobe of the liver, with compression of the stomach, transverse colon and abdominal wall. Biopsy revealed hepatocellular carcinoma with extensive necrosis and ultimately the patient required surgical intervention, which required resection of omentum, partial gastrectomy, hepatectomy, and extensive dissection of the mass to separate the tumor from the remaining intra‐abdominal structures and the retroperitoneum. Peduncular hepatocellular carcinomas vary in presentation and difficulty of resection. The prognosis and probability of successful surgical intervention depends on the level of differentiation, early staging, size of the neoplasm and invasion into surrounding structures. In this case, R0 resection was successfully performed.

Hepatocellular carcinoma is associated with hepatitis viral infection, alcohol use, metabolic syndrome, and non‐alcoholic fatty liver disease. Pedunculated Hepatocellular carcinoma is a rare subtype, which can be further categorized by its presence or absence of a pedicle attaching it to the liver. Its histological classification, as well as its clinical staging, determines patient prognosis. We report a case of pedunculated hepatocellular carcinoma, which required careful pre‐operative planning and extensive surgical intervention to achieve R0 resection.

## 2 CASE PRESENTATION

**FIGURE 1:**
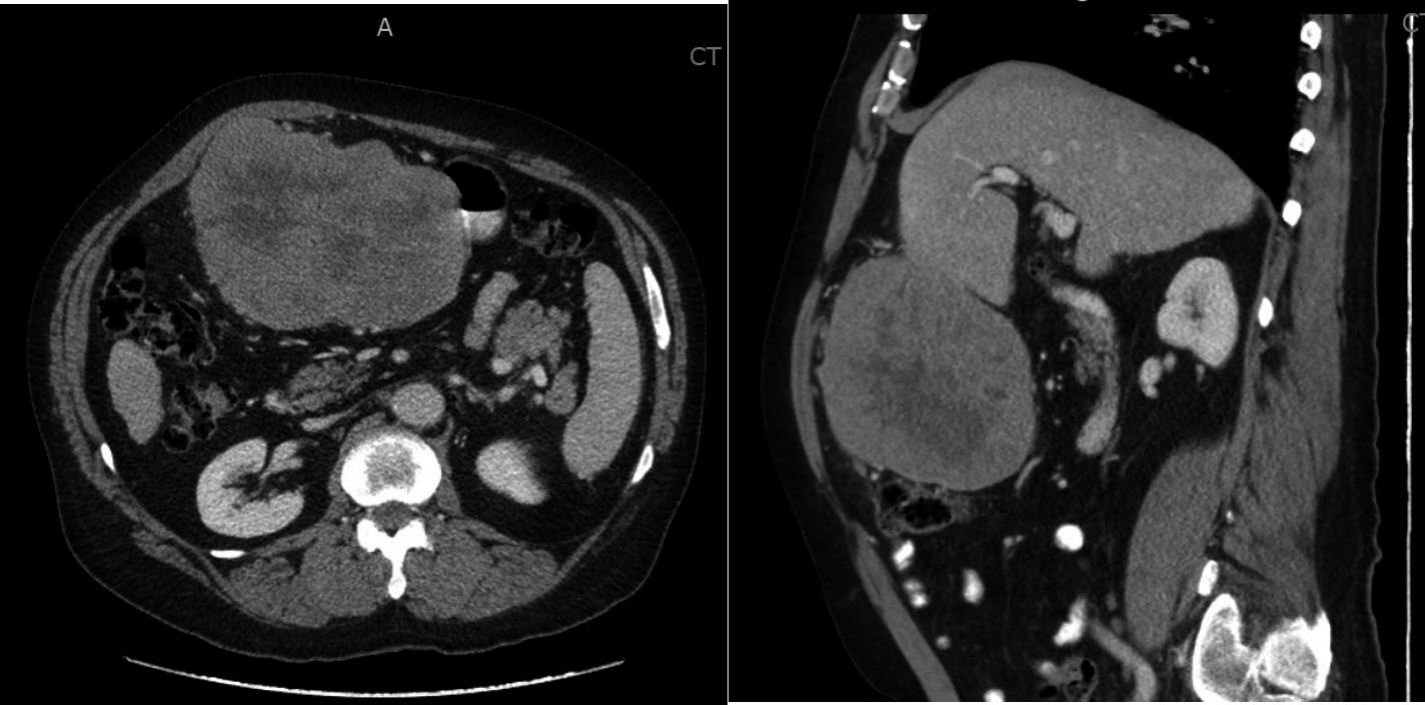
CT scan of the large, pendular mass originating from the left lobe of the liver. Note the hypodense area inside the neoplasm suggestive of necrosis. The left image shows the stomach intimately associated with the mass with transverse colon sitting posteriorly. The image on the right shows the mass compressing the anterior abdominal wall. He is staged as a T3N0M0

A 66‐year‐old male with a history of Hepatitis C treated in 2009 with Ledipasvir/Sofosbuvir (Harvoni^®^) initially presented to the Medical Oncology team in Hackensack University Medical Center, NJ with abdominal discomfort and distention for 45 months associated with an approximate 15 pounds of weight loss. He underwent CT scan of the abdomen with contrast and was found to have a large 15.0 × 11.0 × 13.0 cm heterogenous mass attached to the left lobe of the liver, with compression of surrounding intra‐abdominal contents including the stomach and transverse colon, as well as the abdominal wall (Figure 1). Subsequent percutaneous biopsy was performed for genetic testing, and results demonstrated hepatocellular carcinoma with extensive necrosis. He was then referred to the Surgical Oncology team for further workup and management. Follow up MRI demonstrated two additional satellite lesions consistent with multifocal hepatocellular carcinoma without evidence of invasion into surrounding vital structures. Surgical intervention was deemed necessary given the size of the mass and symptomology. A baseline AFP was 6.1, he was designated as a Child Pugh Score of 8 and he had a MELD Score of 9.

**FIGURE 2:**
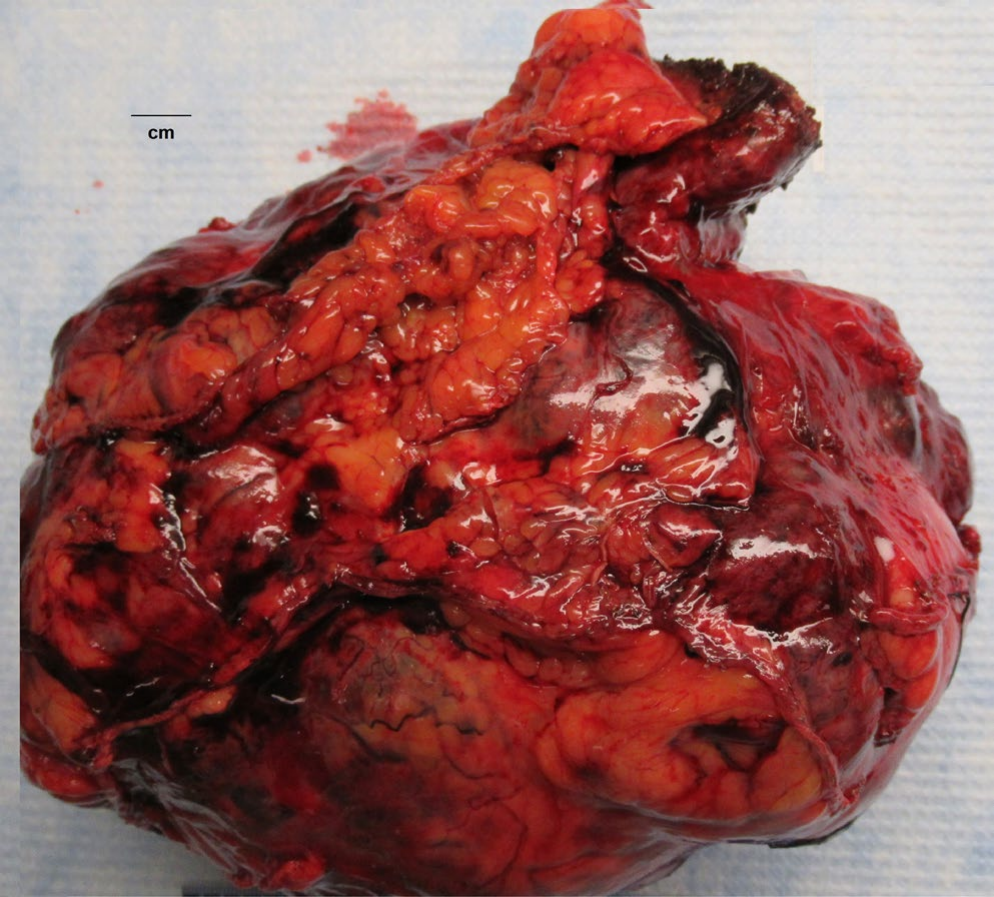
Gross image of the hepatocellular carcinoma. The resected specimen shows a large, lobular tumor grossly

In the operating room, two large‐bore intravenous catheters were placed but fluid was kept at a low rate along with placement of a foley catheter. A laparotomy was performed; making a midline sub‐xiphoid incision, which was extended down to the pubic symphysis due to the size of the tumor (Figure 2). Multiple friable blood vessels were noted at the umbilicus. The omentum as then resected, freeing the anterior abdominal wall. The transverse colon was then carefully separated away from the tumor using a GIA stapler. The transverse colon and its mesentery were preserved and protected from the dense mass. It was also noted that the distal stomach was adhered to the mass along the greater curvature. A sleeve gastrectomy was also completed, requiring three firings for completion.

At this time, the retroperitoneal attachment to the duodenum was carefully dissected, followed by separation of the pancreatic head. This allowed the tumor to be separated from the retroperitoneum and lifted out of the abdomen. A Pringle maneuver was performed using a vascular clamp, and the satellite lesions were transected using parenchymal ablation using the NeuWave Microwave Ablation system. The midline laparotomy was closed in the usual fashion.

**FIGURE 3:**
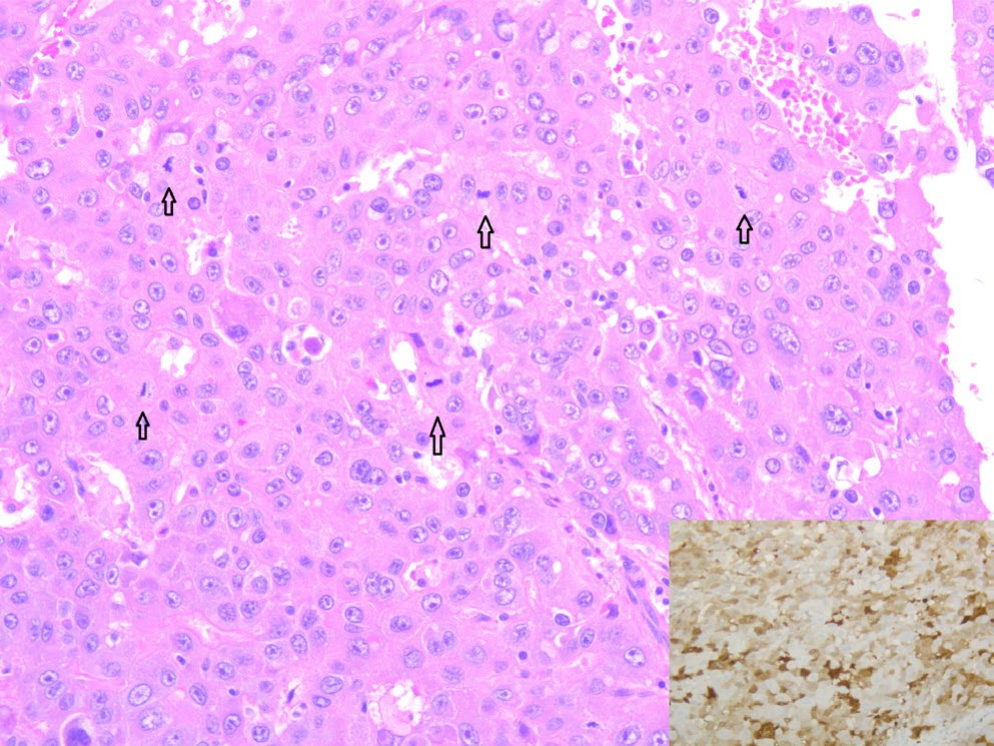
Microscopically, the tumor demonstrates solid and trabecular growth pattern with round nucleus, prominent nucleoli and abundant cytoplasm, which are typical for hepatocellular carcinoma (Hematoxylin and Eosin stain, 200 magnification). Mitotic figures are easy to see (open arrow). Insert shows tumor cells are positive for Arginase on immunostain

**FIGURE 4:**
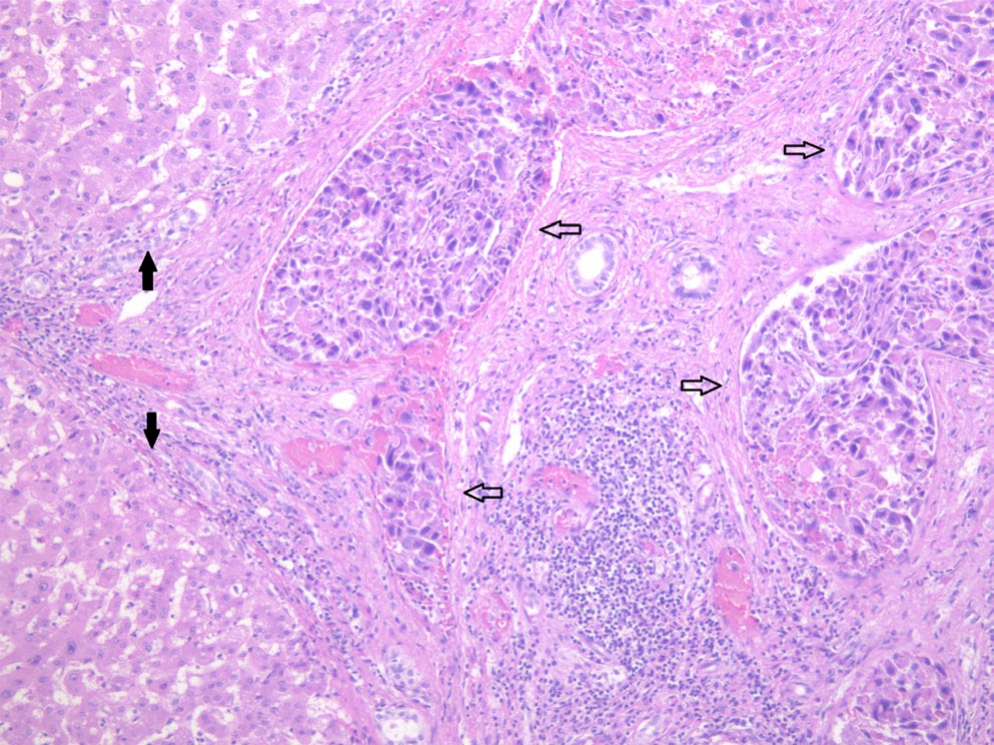
In addition, the tumor shows extensive vascular invasion (open arrow) into the portal veins. The background liver shows well‐established cirrhosis (solid arrow)

On gross inspection, the resected specimen was a 21.0 × 20.0 × 10.7 cm, tan‐yellow mass weighing 1948 g (Figure 2). The tumor is partially adhered to a portion of the stomach and surrounded by adjacent background liver. Microscopically, the tumor shows solid and trabecular growth pattern with round nucleus, prominent nucleoli, abundant cytoplasm and distinct cell border typical for hepatocellular carcinoma (Figure 3). Mitotic figures are frequent and tumor necrosis is also seen. Multiple satellite nodules are seen adjacent to the main tumor and extensive small vessel invasion is present (Figure 4). The tumor also invades into the gastric wall. The background non‐tumor liver shows well‐formed cirrhotic liver with mild to moderate activity. The attached portion of the stomach demonstrated negative margins (R0). The specimen from the partial hepatectomy, which was the satellite lesion of segment 3, demonstrated a similar pattern.

The post‐operative course was significant for urinary retention. Given his history of benign prostate hyperplasia (BPH), a foley catheter was reinserted. He had also developed an ileus requiring a nasogastric tube, which was removed four days later. The patient developed serosanguineous drainage from his incision site after a bout of heavy coughing. A Prevena VAC was placed but was discontinued one week later when the output was reduced to zero, and he was discharged from the hospital later in the day.

## 3 DISCUSSION

Hepatocellular carcinomas (HCC) are aggressive tumors prone to metastasis and make up approximately 75% of primary hepatic malignancies,^1^ with exophytic tumors being one of the rarest presentations. Risk factors include Hepatitis B, Hepatitis C, alcohol use, and non‐alcoholic fatty liver disease (NAFLD), which result in cirrhosis and ultimately develop into HCC.^2^, ^3^, ^4^ It is the most common primary liver malignancy^3^ and the fourth most common cause of cancer‐related death worldwide and its incidence is rising.^5^ Diagnosis requires histological evaluation and staging is primarily based on computed tomography (CT scan) and magnetic resonance imaging (MRI).

Hepatocellular carcinoma is histologically classified into four grades using the Edmonson‐Steiner Classification. Grade I carcinoma includes a mostly‐differentiated tumor which is arranged in a thin trabecular pattern. Grade II shows enlarged nuclei with abundant cytoplasm, with cells maintaining trabecular pattern. Grade III carcinoma demonstrates large, hyperchromatic nuclei with less bile and acinar formation. The hallmark of grade III disease is the abundance of tumor giant cells. Grade IV carcinoma is poorly differentiated, with tumor cells varying in cytoplasmic abundance and quality, along with near absence of bile. These cells are disorganized, and cohesion molecules are often absent.^6^

Pedunculated HCC (P‐HCC) presents as two subtypes either with or without a pedicle.^7^ Moderate or poor differentiation demonstrates an unfavorable prognosis if not intervened upon early, primarily due to their rapidly progressive nature.^8^ When compared to non‐pedunculated neoplasms (NP‐HCC), P‐HCC tumors >5 cm demonstrate poor outcomes. These masses protrude into the abdomen so there is limited growth into adjacent liver parenchyma. As a result, they are more often amenable to curative resection if resected in a timely manner. Presence of a tumor capsule suggests early staging, and tends to play a role in resisting tumor expansion. As the portal vasculature supplies blood to the liver, vascular invasion plays an important role in recurrence.^9^ Patients who present with recurrent disease tend to have peritoneal carcinomatosis, as well as metastasis to the spleen^6^ and adrenal glands.^10^

Hepatocellular carcinomas causes chronic inflammation and persistent cytokine release^11^ along with angiogenesis.^4^ The release of VEGF is responsible for the development of new blood vessels, which are both structurally and functionally abnormal. These vessels are highly permeable and provide poor oxygenation resulting in hyper‐vascular regions with necrosis.^12^

Surgical resection has been shown to be curative and is considered first‐line therapy if R0 resection is possible to achieve. Such interventions depend on the size and location of the tumor, as well as the pre‐operative liver function and these patients require evaluation for post‐resection liver volume.^13^, ^14^ This large pedunculated neoplasm was separate from the left lobe of the liver, which is precisely why its resection was a feasible option. Fluids were provided at a low rate to keep central venous pressure low, to avoid post‐operative bleeding at the liver. The intra‐operative decision to perform a sleeve gastrectomy was because there was concern for the posterior stomach to have positive margins, as it was adhered densely to the neoplasm.

## 4 CONCLUSION

We report a 66‐year‐old male presenting with a large, peduncular intra‐abdominal hepatocellular carcinoma extending into the retroperitoneum with satellite lesions requiring an exploratory laparotomy, resection of the tumor, partial gastrectomy, and hepatectomy. Surgical intervention was ultimately therapeutic despite the technical difficulty of achieving R0 resection.

## CONFLICT OF INTEREST

None declared.

## AUTHOR CONTRIBUTIONS

The DO, JD, XC, HZ and DAM all contributed to the manuscript, review of literature, acquisition of data, analysis and interpretation of data. All authors have given final approval of the version to be published and agree to be accountable for all aspects of the work.
